# DNAAF5 promotes hepatocellular carcinoma malignant progression by recruiting USP39 to improve PFKL protein stability

**DOI:** 10.3389/fonc.2022.1032579

**Published:** 2022-10-06

**Authors:** Yaping Liu, Qiong Wu, Tiantian Sun, Junxing Huang, Gaohua Han, Hexu Han

**Affiliations:** ^1^ Department of Oncology, The Affiliated Taizhou People’s Hospital of Nanjing Medical University, Jiangsu, China; ^2^ Department of Geriatrics, The Affiliated Taizhou People’s Hospital of Nanjing Medical University, Jiangsu, China; ^3^ Medical Department, The Affiliated Taizhou People’s Hospital of Nanjing Medical University, Jiangsu, China; ^4^ Department of Gastroenterology, The Affiliated Taizhou People’s Hospital of Nanjing Medical University, Jiangsu, China

**Keywords:** DNAAF5, glycolysis, prognosis, HCC, PFKL protein

## Abstract

**Purposes:**

Dynein axonemal assembly factor 5 (DNAAF5) is the transcription factor of regulating the cytoskeleton and hydrodynamic protein complex assembly, however, it was not well elucidated in the malignant progression of hepatocellular carcinoma (HCC).

**Methods:**

We investigated the role of DNAAF5 in hepatocellular carcinoma by using multiple groups of clinical tissues combined with data from the TCGA database. Then we overexpressed DNAAF5 in hepatocellular carcinoma tumor tissues, which correlates with poor patient survival outcomes. Furthermore, we constructed stable cell lines of HCC cells to confirm the cancer-promoting effects of DNAAF5 in hepatocellular carcinoma. To explore the mechanisms of DNAAF5, transcriptome sequencing combined with mass spectrometry was also performed, which showed that DNAAF5 affects its downstream signaling pathway by interacting with PFKL and that DNAAF5 regulates PFKL protein stability by recruiting the deubiquitination protein, USP39. To corroborate these findings, the same series of tissue microarrays were used to confirm correlations between DNAAF5 and PFKL expressions. In animal experiments, DNAAF5 also promoted the proliferation of HCC cells.

**Results:**

We found that DNAAF5 expressions were markedly higher in HCC tissues, compared to the adjacent normal tissues. Increased levels of DNAAF5 were associated with significantly worse prognostic outcomes for HCC patients. Cell function experiments showed that HCC cells of overexpressing DNAAF5 exhibited faster proliferation rates, stronger clone formation abilities and higher drug resistance rates. However, tumor cell proliferation rates and colony formation were significantly decreased after DNAAF5 knockout, accompanied by an increase in sensitivity to sorafenib. In addition, the results of our study showed that DNAAF5 accelerates PFKL protein deubiquitination by recruiting USP39 in HCC cells. Furthermore, The overexpression of DNAAF5 could promote HCC cell proliferation *in vivo* and *in vitro*, whereas USP39 knockdown inhibited this effect. Overall, DNAAF5 serves as a scaffold protein to recruit USP39 to form a ternary complex by directly binding the PFKL protein, thereby improving the stability of the latter, which promotes the malignant process of hepatocellular carcinoma.

**Conclusions:**

These findings revealed DNAAF5 was negatively correlated with the prognosis of patients with hepatocellular carcinoma. It underlying mechanism showed that DNAAF5 directly binds PFKL and recruits the deubiquitinated protein (USP39) to improve the stability of the PFKL protein, thus enhancing abnormal glycolysis in HCC cells.

## Introduction

Primary liver cancer is a leading cause of cancer deaths worldwide, accounting for more than 700,000 deaths each year. It is the fourth most common cause of cancer-related death worldwide and ranks third in terms of incidences (1). Primary liver cancer types include hepatocellular carcinoma (HCC), intrahepatic cholangiocarcinoma (ICC) and HCC-ICC mixed type. Among them, HCC accounts for 85% ~ 90% of all cases (2). In this study, our focus is on the pathological type of HCC. Currently, various therapeutic options for HCC, including hepatectomy or liver transplantation, are only suitable for early patients. A large number of advanced patients often lack effective treatment options, resulting in rapid terminal events of the disease (3–5). It is necessary to elucidate the pathogenesis of this disease, with the aim of identifying new therapeutic targets.

Glycolysis, a multi-step process that is mediated by multiple proteins in the membrane and cytoplasm, is regulated by three key enzymes, Hexokinase, PFK and PKM2 (4–6). Variations in functions of these molecules in tumors will promote or inhibit abnormal glycolysis in tumor cells. Among all molecular regulating mechanisms, protein post-translational modification (PTM) is an important way to regulate protein functions. Under physiological and pathological conditions, various types of protein PTMs in cells can affect the activity, stability, localization and signal transduction of proteins, which can rapidly regulate various life activities, thereby expanding the diversity of protein functions (7). Ubiquitination is an important type of post-translational protein modification. It plays important role in the malignant processes of HCC and affects therapeutic efficacies (8). However, studies on the Warburg effect of ubiquitination, especially on the regulation of ubiquitination of the three key enzymes are not conclusive.

Dynein axonemal assembly factor 5 (DNAAF5) is also known as ciliary dyskinesia-18 (CILD18) or heat repeat-containing protein 2 gene (HEATR2). Studies on this molecule are very limited, with whole-exome sequencing in 2012 identifying HEATR2 mutations as the cause of primary ciliary dyskinesia (9). In 2014, it was reported that HEATR2 has a conserved role in ciliary motor assembly (10), and in 2018, Amjad Horani et al. reported that HEATR2 is an early scaffold for the assembly of dynein complexes in motile cilia (11). These findings imply that studies on this molecule are limited to its role in the cytoskeleton and cytodynamic protein complex assembly, while its biological roles in malignant tumor pathogenesis, especially HCC, have not been reported. RNA-based expert annotation of TCGA gave inconclusive results showing that DNAAF5 could be highly expressed in liver cancer and head and neck cancer, but it still needs further experimental verification.

In this study, we evaluated the mechanisms of DNAAF5 in HCC and found that DNAAF5 is generally highly expressed in tumor tissues of HCC patients, compared to their adjacent tissues. These elevated levels were negatively correlated with HCC patient prognosis. Furthermore, DNAAF5, as a scaffold protein, recruited USP39 to form a ternary complex by directly binding the PFKL protein, thereby improving the stability of the latter. These data indicated that DNAAF5 is a good prognostic marker for HCC and a potential therapeutic target.

## Materials and methods

### Cell cultures and treatments

The HCC cell line and normal liver cell lines (Lo2) were purchased from Shanghai Fuheng Technology Co., Ltd. and cultured in the corresponding medium supplemented with 10% fetal bovine serum (FBS) and 1% penicillin/streptomycin (Invitrogen). Incubation and passaging were performed according to standard protocols in a humidified atmosphere containing 5% CO_2_ at 37°C (12).

### CCK8 cell proliferation assay

All cells were planted in 96-well plates at a density of 3000 cells/well and cultured for 24 h. Thereafter, the cells were transfected with DNAAF5 plasmid (60 nM), negative control siRNA for 24, 48, 72 or 96 h, respectively. Each well was incubated with 10 μg Cell Counting Kit-8 solution (Dojindo, Shanghai, China) for 4 h. Cell proliferation was estimated by examining the absorbance at 450 nm with Varioskan Flash Spectral Scanning Multimode Reader (Waltham, MA, USA).

### Gemcitabine killing assay

After collecting all cells, viability dye 7-Aminoactinomycin D (7-AAD, Sigma, A9400) was measured on the FC500. Then 10 µL Flow-Count Fluorosphere Beads (Beckman Coulter, 7547053) were added, and the number of viable cells was analyzed. Percentage of gemcitabine-mediated death was calculated as follows: (1 – (number of viable cells treated with gemcitabine)/(number of viable cells treated without gemcitabine)) × 100%.

### RNA extraction and qPCR assays

Total RNA extraction from cells was performed using the RNA-easy Isolation Reagent (Vazyme Biotech Co., Ltd). Then, 1 ug RNA was reverse transcribed using the HiScript III 1^st^ Strand cDNA Synthesis Kit (+gDNA wiper) (Vazyme Biotech Co., Ltd). Quantitative real-time (qRT) PCR was performed using AceQ Universal SYBR qPCR Master Mix (Vazyme Biotech Co., Ltd) in the ABI 7500 Detection System (Applied Biosystems). The extracted total RNA was sent to Shanghai Jingfang Company for transcriptome sequencing and analysis.

Primer sequences of related genes were:

RT-B-actin-F: TCCCTGGAGAAGAGCTACG
RT-B-actin-R: GTAGTTTCGTGGATGCCACA
RT-DNAAF5-F: CAGTCGGAGTCTCTGATCGG
RT-DNAAF5-R: CAAACAGTCGCTGAGCAAAATG
RT-PFKL-F: GCTGGGCGGCACTATCATT
RT-PFKL-R: TCAGGTGCGAGTAGGTCCG

### Plasmid construction

We constructed overexpression vectors for DNAAF5, PFKL and USP39 genes using pCDNA3.1-puro-Flag, pCDNA3.1-G418-myc and pCDNA3.1-Hygro-HA plasmids, respectively. The extracted RNA was reverse transcribed to cDNA using the HiScript III 1^st^ Strand cDNA Synthesis Kit (+gDNA wiper) (Vazyme Biotech Co., Ltd), after which the corresponding fragment was amplified using 2 × Phanta Flash Master Mix (Dye Plus) (Vazyme Biotech Co., Ltd) and a ClonExpress II One The Step Cloning Kit (Vazyme Biotech Co., Ltd) to ligate the fragment to the corresponding vector. Complete plasmids were used for subsequent experiments after sequences had been verified to be correct.

ov-DNAAF5-F: ATGGCGGCGCTGGGGGTG
ov-DNAAF5-R: CTGTGTGGCTGGCACGGCCTG
ov-PFKL-F: ATGGCCGCGGTGGACCT
ov-PFKL-R: GAAGCCCTTGTCCATGCTCA
ov-USP39-F: ATGTCCGGCCGGTCTAAGC
ov-USP39-R: AGCCCCCTGCTGGTTGGT

The LentiCRISPRv2 plasmid was digested using Esp3I (R0734, NEB), after which the annealed primer fragment was ligated into it using T4 ligase (M0202, NEB).

sg-DNAAF5: cggccccctcagccgggtgtg

The PLKO.1-Hygro vector was used to silence the corresponding gene. After double-digestion with AgeI-HF (R3552, NEB) and EcoRI-HF (R3101, NEB), T4 ligase (M0202, NEB) was used to link the annealed primer fragments into the vector.

sh-USP39-1: GTTGCCTCCATATCTAATCTT
sh-USP39-1: CCTTCCAGACAACTATGAGAT

Depending on the progress and needs of the experiment, the Lipo8000 (C0533, Beyotime) transfection reagent was used to transfer different plasmids into various cells after which corresponding antibiotics were added to screen and obtain equivalent stable cell lines.

### Protein extraction and western blotting assays

Cell samples and fresh tissue samples were treated using the RIPA lysis solution (supplemented with protease and phosphatase inhibitors, Vazyme Biotech Co., Ltd), and the supernatant was centrifuged, loaded and boiled. About 30 ug total proteins were separated on sodium dodecyl sulfate-polyacrylamide gel electrophoresis (SDS-PAGE), transferred onto polyvinylidene difluoride (PVDF) membranes, blocked using 5% non-fat milk at room temperature for 2 h, and incubated in the presence of primary antibodies. The membrane was washed thrice and incubated with horseradish peroxidase (HRP)-conjugated secondary antibodies (1: 1000 dilution) for 1 h at room temperature. For protein half-life analysis, cells were treated with CHX (50 ug/ml) at different times. The antibodies used in this assay were: Anti-HK2 (#2867, CST), Anti-PKM2 (#4053, CST), Anti-myc (#9402, CST), Anti-p53 (#2527, CST), Anti-HIF1a (#5537, CST), Anti-PFKL (A7708, Abclonal), Anti-USP39 (23865-1-AP, Proteintech), Anti-SIX1 (10709-1-AP, Proteintech), Anti-DNAAF5 (24578-1-AP, Proteintech), Anti-myc-tag (16286-1-AP, Proteintech), Anti-Flag-tag (F1804, Sigma), Anti-HA-tag (sc-7392, Santa cruz).

### Co-immunoprecipitation assays

The Co-immunoprecipitation kit from Thermo Scientific (26149) was used in this assay, as instructed by the manufacturer. All cell supernatants containing 1–4 mg protein (in ~ 1 mL lysate) was pre-cleared (PC) for 1–4 h at 4 °C with 5–10 μg of suitable mouse antibody isotypes, rabbit immunoglobulins or goat immunoglobulins, and 80–100 μl of Dynabead^®^ protein G (Thermo Fisher Scientific). Precleared supernatants were incubated (overnight, 4 °C) with 5–10 μg of desired IP antibody and 80–100 μl of Dynabead^®^ protein G. Dynabead^®^ Protein G beads bound to control antibody isotypes (i.e., PC complexes) or desired primary antibodies (i.e., IP complexes) were washed for 5 times with IP buffer or wash buffer supplied by the vendor (Thermo Fisher Scientific) and eluted with NuPAGE^®^ LDS Sample Buffer (Thermo Fisher Scientific) in the presence of NuPAGE^®^ Sample Reducing Agent (Thermo Fisher Scientific).

### Pull-down assays

Corresponding kits from Thermo Scientific (21516, 21277) were used in this assay, as instructed by the manufacturer. All cells were treated with 100 mM NaAc and 5 mM sodium (meta)periodate in 200 μl of water and rotated for 1 h at room temperature in the dark. The RNA was precipitated by adding 600 μl of 100% ethanol and 15 μl of 3 M NaAc in dry ice for 20 min, followed by centrifugation at 13 000 rpm, 4°C for 10 min. The RNA pellet was washed with 70% ethanol and resuspended in 500 μl of 100 mM NaAc pH 5. 200 μl of adipic acid dihydrazide-agarose (Sigma-Aldrich) was washed with 100 mM NaAc and mixed with 500 μl of the periodate oxidized pri-miR-7–1-CTL overnight at 4°C in the dark. The pri-miR-7–1-CTL-beads were washed by 2M KCl, Buffer G (20 mM Tris-HCl pH 7.5, 137 mM NaCl, 1 mM EDTA, 1% Triton X-100, 10% glycerol, 1.5 mM MgCl_2_, 1 mM DTT and 200 μM PMSF) and Roeder D, respectively.

### Tumorigenesis assays in nude mice

Male BALB/c athymic nude mice (4 week-old) were obtained from the laboratory animal center of Nantong University, maintained under standard conditions (SPF levels) and cared for in accordance with institutional guidelines. All animal assays were approved by the Institutional Animal Care and Use Committee of Nantong University. Then, to establish subcutaneous neoplasia mice models, 1 × 10^6^ Huh-7 cells in 100 μL of physiological saline were subcutaneously injected into nude mice. After six weeks, subcutaneous tumors were excised for immunohistochemistry (IHC).

### Immunohistochemical staining assays

All slides were incubated at 60°C for 20 min, dewaxed in xylene and rehydrated in gradient ethanol. Then, slides were incubated with 3% hydrogen peroxide for 10 min, followed by antigen recovery using 0.01 M citrate buffer (pH 6.0) for 30 min. After sealing with 5% BSA, slides were incubated overnight in the presence of appropriate primary antibodies at 4°C and thereafter with biotin-coupled secondary antibody at room temperature for 1 h. Using diaminobenzidine reaction visualization IHC staining, redye with hematoxylin.

### Statistical analysis

The GraphPad Prism7 software was used for analyses. Differences in means between and among groups were assessed by the student’s t-test or one-way ANOVA. Statistical differences between different treatments, cell cohorts, or time points were assessed by two-way ANOVA. Overall survival was estimated by the Kaplan-Meier method, and significance was determined by the log-rank test. The intensity of DNAAF5 staining between PFKL was quantified using Image-Pro Plus6, and their correlations were analyzed using the Chi-square test (Fisher’s exact). *p < 0.05, **p < 0.01, ***p < 0.001 were the thresholds for statistical significance; ns represents no statistical significance.

## Results

### Elevated DNAAF5 levels in HCC negatively correlated with prognosis

**Figure 1:**
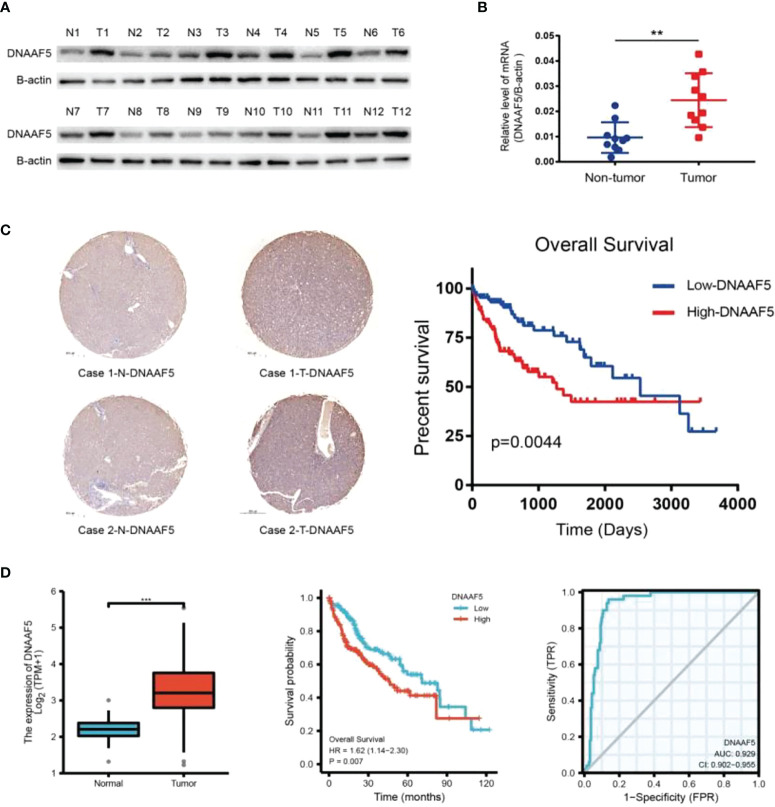
DNAAF5 is highly expressed in hepatocellular carcinoma and negatively correlates with prognosis. **(A)** Expression of DNAAF5 in 12 paired human HCC tumor tissues (T) and matched paracancerous tissues (N) as determined by western blotting assays. **(B)** The mRNA expression of DNAAF5 in hepatocellular carcinoma tissues and in non-tumor tissues adjacent to the cancer as determined using qPCR assays. **(C)** IHC staining results showing the expression of DNAAF5 in cancer tissue and adjacent non-cancerous tissues. Representative staining images of DNAAF5 are shown (left), and high DNAAF5 expression was negatively correlated with patient prognosis (right). **(D)** Analysis of sequencing data of hepatocellular carcinoma in TCGA database indicating higher expression of DNAAF5 in tumor tissues compared to adjacent non-cancerous tissues (left). The expression of DNAAF5 in hepatocellular carcinoma was negatively correlated with patient prognosis (middle). ROC curve (AUG>0.9) illustrating the accuracy of DNAAF5 in predicting patient prognosis (right). **p < 0.01, ***p < 0.001 were the thresholds for statistical significance.

To begin with, we found that DNAAF5 expressions were markedly elevated in HCC tissues, compared to the adjacent normal tissues (**Figure 1A**). In the other cohort of paired tissues, similar outcomes in expressions of DNAAF5 were obtained by real-time quantitative PCR (qPCR) (**Figure 1B**). To investigate the significance of DNAAF5 in HCC prognosis, immunohistochemical staining (IHC) was performed to analyze the tissue chip of paraffin-embedded HCC tissues. Elevated levels of DNAAF5 were associated with significantly worse prognostic outcomes for HCC patients (**Figure 1C**). By analyzing the data of related patients in the TCGA database and combining it with the ROC curve table (AUG>0.9), we concluded that DNAAF5 is a potential prognostic biomarker and involves the disease pathogenesis for HCC patients (**Figure 1D**).

### DNAAF5 promotes multiple malignant phenotype in HCC cells

**Figure 2:**
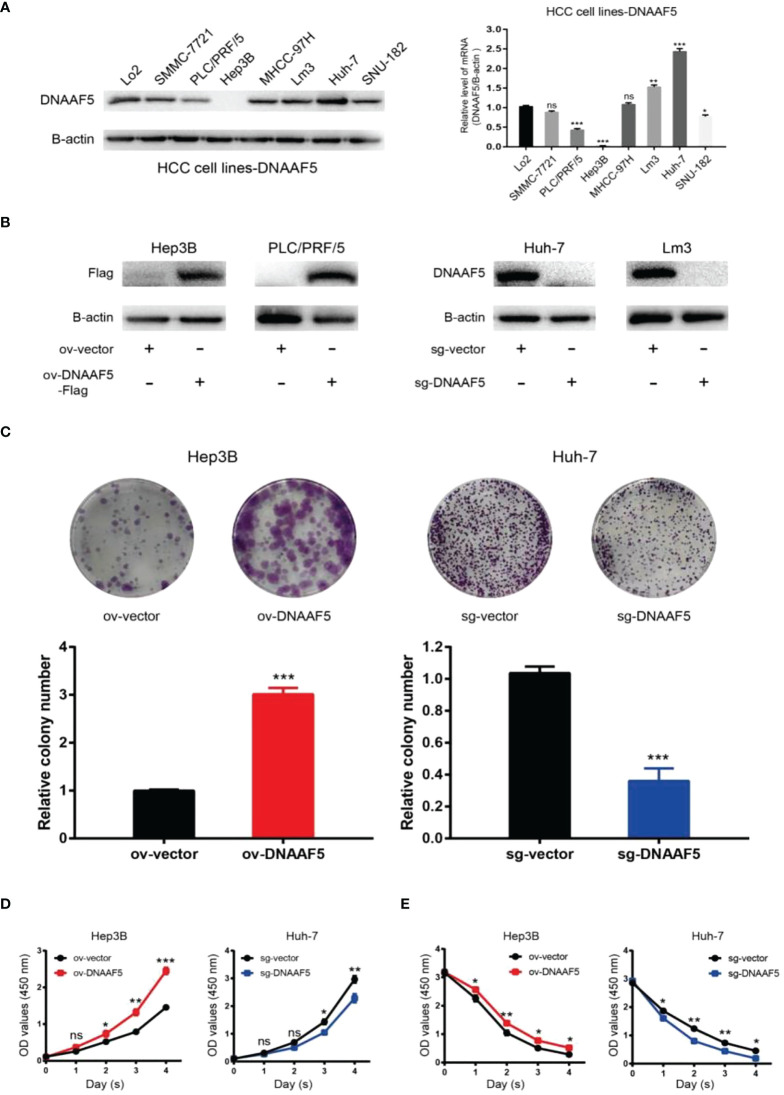
DNAAF5 promotes multiple malignant phenotypes in hepatocellular carcinoma cells. **(A)** Expression level of DNAAF5 in different HCC cell lines as determined by western blotting assays (left), qPCR results showing mRNA level of DNAAF5 in different HCC cell lines (right). **(B)** The expression of DNAAF5 reflecting the successful construction of stable cell lines as determined by western blotting assays. **(C–E)** Results of plate cloning assays **(C)**, CCK8 assays **(D)**, and sorafenib killing experiments **(E)**, showed that DNAAF5 overexpression in HCC cells increased the degree of malignancy and vice versa. *p < 0.05, **p < 0.01, ***p < 0.001 were the thresholds for statistical significance; ns represents no statistical significance.

To further carry out the corresponding exploration process, we performed western blotting and qPCR assays on different HCC cell lines. It revealed that DNAAF5 levels in Huh-7 cells were highest while those in PLC/PRF/5 cells were lowest. In Hep3B cells. DNAAF5 levels could not be detected (**Figure 2A**). Based on these findings, we constructed corresponding stable cell lines. Using the CRISPR-Cas9 technology, DNAAF5 was knocked out in Huh-7 and Lm3 cells, while it was overexpressed in Hep3B and PLC/PRF/5 cells (**Figure 2B**). Using the above-constructed stably transfected cell lines, biological functions of DNAAF5 were investigated. Cell function experiments showed that HCC cells of overexpressing DNAAF5 exhibited faster proliferation rates, stronger clone formation abilities and higher drug resistance rates. However, tumor cell proliferation rates and colony formation were significantly decreased after DNAAF5 knockout, accompanied by an increase in sensitivity to sorafenib. These findings implied that DNAAF5 might play important roles in malignant processes of HCC (**Figures 2C–E**).

### DNAAF5 can activate multiple signaling pathways in HCC

**Figure 3:**
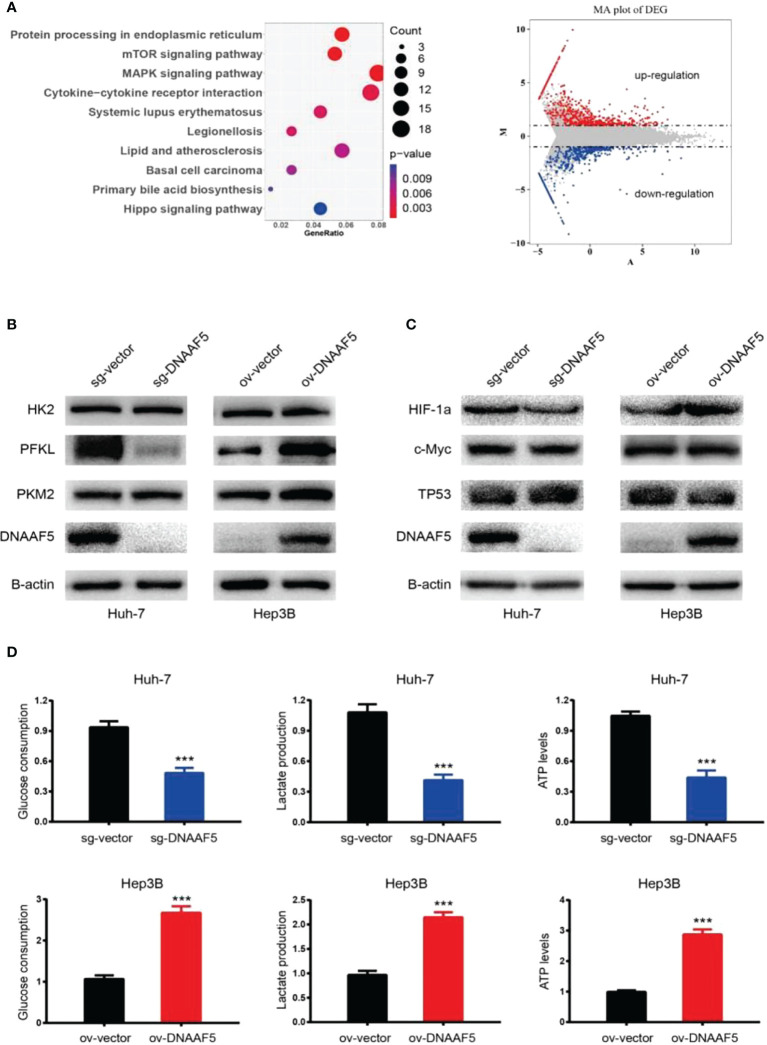
DNAAF5 regulate multiple signaling pathways in hepatocellular carcinoma. **(A)** Transcriptome sequencing results showed that DNAAF5 might regulate multiple signaling pathways (left) and affect the expression of a large number of genes (right). **(B)** The expression level of key enzymes involved in glycolysis as determined by western blotting assays. The expression PFKL was positively associated with expression level of DNAFF5, but the expression level of DNAAF5 did not affect the expression of HK2 and PKM2. **(C)** Intracellular expression level of three key transcription factors (p53, myc and HIF-1a) involved glycolysis was not altered significantly as revealed by western blotting assays. **(D)** Cytological results showing that overexpression of DNAAF5 increased glucose consumption and lactate production, and vice versa. ***p < 0.001.

To investigate the mechanisms of DNAAF5 in HCC, transcriptome sequencing was performed using the constructed Huh-7-sg-vector/DNAAF5 stably transfected cell lines. Sequencing analysis revealed that DNAAF5 affects various biological behaviors which are involved in mediating multiple signaling pathways (**Figure 3A** and **Figure S1A**). To ulteriorly evaluate the mechanisms of action of DNAAF5, co-immunoprecipitation (Co-IP) assays and mass spectrometry were performed to analyze interacting proteins of DNAAF5. It was revealed that DNAAF5 potentially interacts with PFKL (phosphofructokinase, liver type) protein, which encodes the pivotal enzyme that catalyzes the conversion of D-fructose 6-phosphate to D-fructose 1,6-bisphosphate, key step glycolysis, which led us to explore whether DNAAF5 plays an important role in affecting glycolysis, the key biological process in hepatocellular carcinoma, by interacting with PFKL. Western blotting assays revealed that DNAAF5 affects PFKL protein levels in HCC cells, without affecting the other two key enzymes involved in glycolysis (Hexokinase II and PKM2) (**Figure 3B**). However, changes in expressions of the main transcription factors (p53, myc and HIF-1a) that affect PFKL expressions were not marked (**Figure 3C**). Glucose metabolism-related experiments showed that ATP levels, glucose uptake and lactic acid production rates were markedly increased by DNAAF5 overexpression, while related indices were significantly decreased after DNAAF5 knockout (**Figure 3D**). So far, we conjectured that DNAAF5 exerts its cancer-promoting effects by interacting with PFKL, which promotes the Warburg effect and accelerates the malignant processes of HCC.

### DNAAF5 improves the stability of PFKL protein

**Figure 4:**
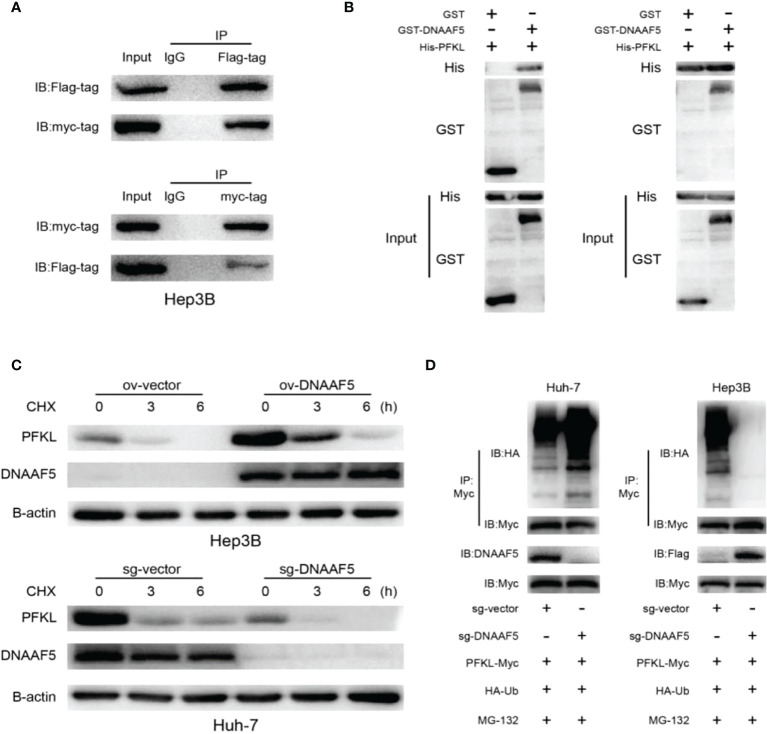
DNAAF5 improves the stability of PFKL protein. **(A)** The interaction between DNAAF5 and PFKL proteins in Hep3B cells as determined using immunoprecipitation (IP) followed by immunoblotting. IgG was used as a negative control for immunoprecipitation. **(B)** Protein purification and pulldown results demonstrating direct interaction between DNAAF5 and PFKL. Results of GST- and His-pulldown experiments indicating that only GST-DNAAF5 directly interacted with His-PFKL, but not GST protein. **(C)** DNAAF5 overexpression significantly improved protein stability of PFKL in Hep3B cells, but knockout DNAAF5 enhanced the degradation of PFKL in Huh-7 cells (cells were exposed to cycloheximide (CHX 50 mg/ml) for 0, 3 or 6 h). **(D)** DNAAF5 overexpression significantly attenuated the ubiquitination of PFKL, while DNAAF5 knockdown significantly enhanced the ubiquitination of PFKL in HCC cells (cells were treated with MG132 (10 mM) for 6 h).

To verify the proposed hypothesis, co-immunoprecipitation (Co-IP) assays were performed using Hep3B cells. Experimental results verified potential interactions between DNAAF5 and PFKL proteins (**Figure 4A**), while pull-down assays confirmed that DNAAF5 directly binds PFKL proteins (**Figure 4B**). Interestingly, qPCR results showed that changes in PFKL mRNA levels after DNAAF5 knockdown/overexpression in HCC cell lines were not significant (**Figure S1B**). Thus, we evaluated whether DNAAF5 affects its intracellular levels by influencing PFKL protein stability. After the treatment of protein synthesis inhibitor (CHX), the degradation rate of PFKL proteins was significantly accelerated for DNAAF5 knockout in Huh-7 cells. On the contrary, PFKL protein stability was significantly elevated by overexpressing DNAAF5 in Hep3B cells (**Figure 4C**). When the constructed stable cell lines were treated with the proteasome inhibitor (MG-132), we found that the polyubiquitination chain of the PFKL protein was significantly enhanced after DNAAF5 deletion in Huh-7 cells, whereas the polyubiquitination chains of the PFKL protein were sharply attenuated upon overexpression of DNAAF5 in Hep3B cells (**Figure 4D**). These findings indicate that DNAAF5 promotes the malignant processes of HCC by regulating PFKL protein stability. Collectively, these findings indicate that DNAAF5 enhances PFKL protein stability by directly interacting with PFKL, thereby inhibiting the formation of polyubiquitination chains on the PFKL protein to elevate its intracellular levels.

### DNAAF5 accelerates PFKL protein deubiquitination by recruiting USP39

**Figure 5:**
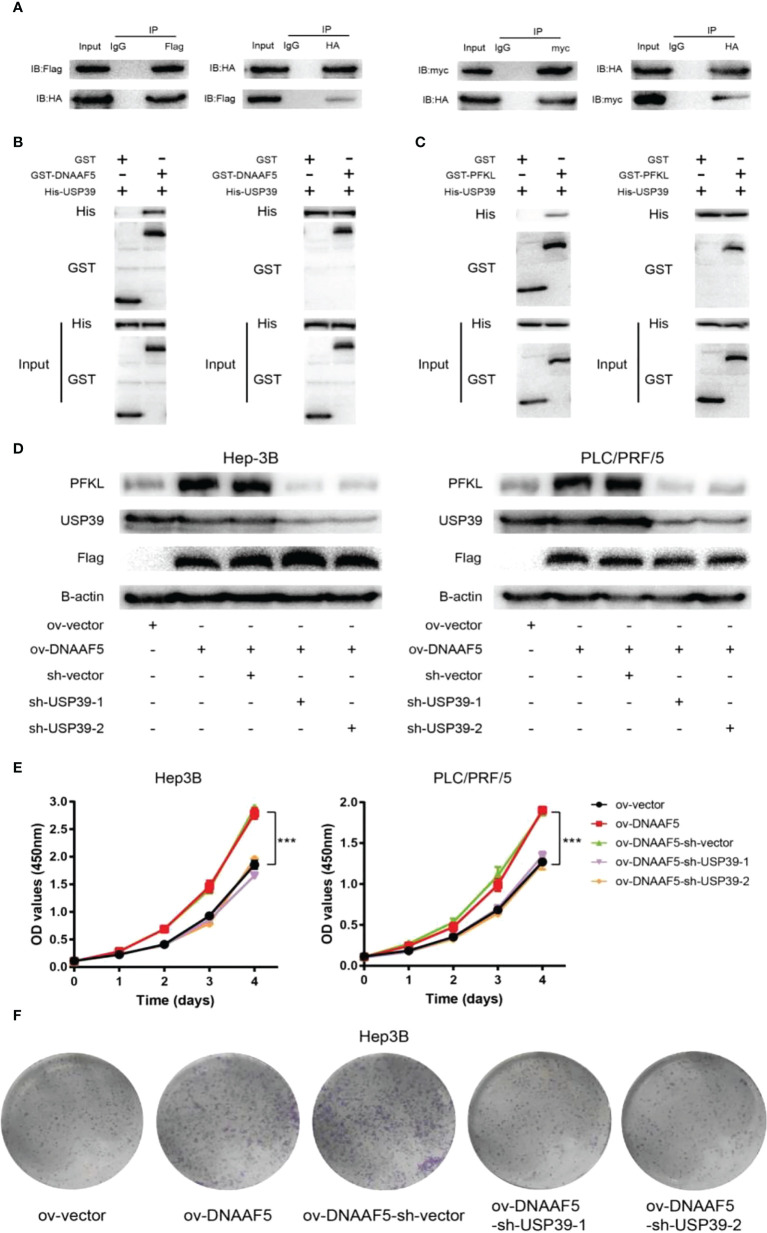
DNAAF5 accelerates the deubiquitination of PFKL protein by recruiting USP39. **(A)** The interaction between DNAAF5-PFKL and PFKL-USP39 proteins in Hep3B cells as determined using immunoprecipitation (IP) followed by immunoblotting. IgG served as the negative control for immunoprecipitation. **(B)** Protein purification and pulldown assays confirming the direct interaction between DNAAF5 and USP39. The GST- and His-pulldown experiments demonstrated that only GST-DNAAF5 directly interacted with His-USP39, but not GST protein. **(C)** Protein purification and pulldown assays results demonstrating the direct interaction between PFKL and USP39. The GST- and His-pulldown experiments showed that only GST-PFKL directly interacted with His-USP39, but not GST protein. **(D)** Upregulation of PFKL expression caused by changes in DNAAF5 expression was rescued by knock down of USP39 in Huh-7 and PLC/PRF-5 cells. **(E)** The CCK8 assays showed that DNAAF5 overexpression promoted the proliferation of tumor cells, whereas USP39 knockdown inhibited this effect in HCC cells. **(F)** The plate cloning assays showed that DNAAF5 overexpression promoted the growth of tumor cells, whereas USP39 knockdown inhibited this effect in Hep3B cells. ***p < 0.001.

Analysis of the amino acid sequence of DNAAF5 revealed that there is no classical known domain that can mediate deubiquitination, which arouses our further thinking. Combined with mass spectrometry results above, we found that the deubiquitination protein (USP39) was among the proteins with potential interactions with DNAAF5. Based on this clue, interactions between DNAAF5-USP39 and USP39-PFKL were confirmed by co-immunoprecipitation assays in Hep3B cells (**Figure 5A**). The GST-pulldown and His-pulldown assays further confirmed that DNAAF5/USP39/PFKL forms heterotrimers *via* direct interactions between the two, implying that DNAAF5 stabilizes the PFKL protein by recruiting USP39 (**Figures 5B, C**). To this end, we designed and constructed two different small hairpin RNAs targeting different sites of USP39, and respectively transferred them into PLC/PRF/5/Hep3B-ov-DNAAF5 cells. Western blot assays showed that USP39 knockdown in PLC/PRF/5/Hep3B-ov-DNAAF5 cells suppressed the increase in PFKL expressions that were a result of DNAAF5 overexpression (**Figure 5D**). To verify the significance of the above cellular and molecular experimental findings on HCC cell proliferation *in vivo and in vitro*, the CCK8 assays and plate cloning assays were performed in HCC cell lines, and the resuls showed that DNAAF5 overexpression promoted the proliferation of tumor cells *in vitro*, whereas USP39 knockdown inhibited this effect (**Figures 5E, F**). Taken together, these molecular and cytological assays showed that DNAAF5 recruits USP39 and PFKL by acting as a scaffold protein, and enhances PFKL protein stability in the form of deubiquitination through interactions between USP39 and PFKL, which accelerates glycolysis to promote the malignant processes of HCC.

### The overexpression of DNAAF5 could promote HCC cell proliferation *in vivo* and *in vitro*, whereas USP39 knockdown inhibited this effect

**Figure 6:**
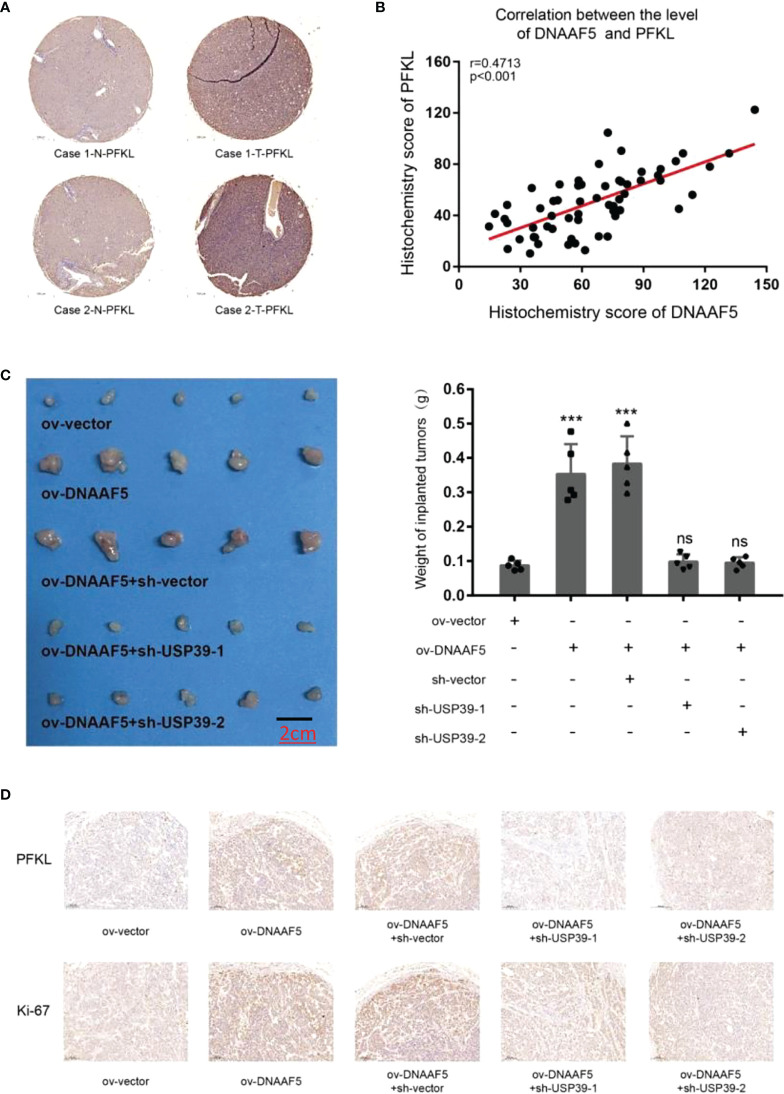
DNAAF5 promotes the growth of hepatocellular carcinoma *in vivo* **(A)** IHC staining indicating a higher expression of PFKL in cancer samples compare to adjacent non-cancerous tissues, the chip shown is from the same group as in **Figure 1**, and the same location was selected. PFKL expression gradually increased with increase in DNAAF5 expression, indicating strong correlation between DNAAF5 and Warburg effect. **(B)** The statistical results of immunohistochemical staining showing a significant positive correlation between DNAAF5 expression and PFKL expression level. **(C)** DNAAF5 overexpression promoted the growth of tumor xenografts in nude mice, whereas USP39 knockdown inhibited this effect. The left panel shows photographs of the tumors and the right panel shows the weight of tumors (scale bar: 2cm). **(D)** The immunohistochemical staining assays showed that DNAAF5 overexpression promoted the tumor proliferation *in vivo* assays, whereas USP39 knockdown inhibited this effect (scale bar: 10μm). ***p < 0.001; ns represents no statistical significance.

We further explored the clinical value of the DNAAF5-PFKL pathway in the malignant progression of HCC. In **Figure 1**, we used tissue microarray to analyze the expressions of DNAAF5 in tissues and its relationship with prognostic outcomes. We applied the same series of the above-mentioned tissue chips and applied immunohistochemical staining assays (IHC) to assess the expressions of PFKL proteins in cancer and adjacent tissues. Then, we analyzed its relationship with DNAAF5 expressions. Compared with non-tumor tissues, PFKL protein levels in tumor tissues were generally high (**Figure 6A**). There was an obvious positive correlation between DNAAF5 and PFKL levels (**Figure 6B**), which verified the results obtained in the above molecular experiments, but also reflected the clinical significance of DNAAF5/PFKL axis in HCC. In addition, *In vivo* tumor formation assays showed that elevated levels of DNAAF5 promote HCC cell proliferation rates, and after USP39 knockdown in Hep3B-ov-DNAAF5 cells, the increased tumor volume due to increased expressions of DNAAF5 was significantly decreased (**Figure 6C**). Findings from the tumor proliferation *in vivo* assays were further verified by immunohistochemical staining assays. This result was corroborated by the molecular and cytological experiments above (**Figure 6D**).

## Discussion

Globally, HCC is one of the most common malignancies and the third leading cause of tumor associated deaths (13). Biologically, HCC cells exhibit high rates of aerobic glycolysis, a hallmark of cancer cells. Elevated aerobic glycolysis levels are associated with significantly high glucose consumption and lactate production rates as well as high ATP levels. This metabolic reprogramming gives HCC cells a growth advantage by providing energy for cancer cell growth and other metabolic intermediates for various processes (14). As a multi-step process, its progress is tightly regulated by three key rate-limiting enzymes (Hexokinase, PFK and PKM2) (15). In this study, we established that the highly expressed DNAAF5 proteins in HCC promote the malignant progression of tumor cells by increasing PFKL protein levels.

In 1920, the German physiologist Warburg, discovered that glycolytic activities in liver cancer cells are more active than those of normal liver cells. Theoretically, this implies that even under aerobic conditions, tumor cells preferentially perform glycolysis rather than the productive oxidative phosphorylation pathway. This effect is manifested by a high rate of glucose uptake, active glycolysis, and high lactate levels (15). Tumor cells also use this special metabolic method to obtain energy and materials for building cellular structures, enhancing malignant cell proliferation. To maintain homeostasis, cells must respond to rapid changes in internal and external environments promptly. Post-translational modifications (PTM) of specific proteins is an extremely sensitive, rapid and reversible regulation method (16). Among them, ubiquitination is a major modification process. In tumor progression, it is often accompanied by imbalances of intracellular ubiquitination and deubiquitination, which is often accompanied by the activation of pro-cancer pathways and suppression of anti-cancer pathways (17). In glycolysis, CSN5 functions as a deubiquitinating enzyme, attenuating the degradation of HK2 by proteasomes, thereby enhancing glycolysis and metastasis of HCC cells (18). However, the regulation of PKM2 by ubiquitination-related processes is more diverse. For instance, U-box E3 ligase CHIP reduces aerobic glycolysis by ubiquitylating and degrading PKM2 in ovarian carcinoma (OV) cells, while CTLH ubiquitylates PKM2 and lactate dehydrogenase A (LDHA) in a non-degradative pathway, thereby inhibiting glycolysis (19). On the contrary, USP7/USP20 increases the stability of PKM2 proteins by directly interacting with PKM2 (20). Relevant studies on PFKL ubiquitination are relatively scarce, only one study has reported that the E3 ligase, A20, can target PFKL for ubiquitination and degradation, thereby inhibiting glycolysis in HCC (21). But in this study, we found that the key rate-limiting enzyme (PFKL) involved in glycolysis was recruited together with the deubiquitination protein USP39 to the highly expressed scaffold protein, DNAAF5, in HCC. This abnormally enhanced deubiquitination process greatly improved the protein stability of PFKL, which promoted the malignant progression of HCC.

However, this study is associated with various limitations. First, the results of our multiple cohorts showed high DNAAF5 expressions in HCC, however, it is not clear how the expressions of this molecule are regulated in cells. Second, The results of this study show that DNAAF5 improves intracellular PFKL levels by recruiting the deubiquitinated protein USP39. However, transcriptome sequencing showed that DNAAF5 is potentially involved in the regulation of multiple signaling pathways, such as the mTOR signaling pathway, MAPK signaling pathway, as well as Hippo signaling pathway, which was not verified whether it is involved in these signaling pathways and its intrinsic mechanisms of action are unclear, at the same time, these results implied that DNAAF5 might mediate related processes of gene transcription in addition to participating in the post-translational modification of proteins. Finally, cell level assays showed that DNAAF5 knockout markedly inhibited HCC cell proliferation as well as migration and improved cell drug sensitivity, suggesting that DNAAF5 has a potential value for clinical translation.

In conclusion, we first found that DNAAF5 was highly expressed in HCC tissues through multiple groups of clinical samples and the TCGA database, and elevated DNAAF5 levels negatively correlated with prognostic outcomes for HCC patients. Then, through various cell biology and molecular biology experiments, it was established that DNAAF5 can be used as a scaffold protein to recruit the deubiquitination protein (USP39) and the rate-limiting enzyme (PFKL) of glucose metabolism to improve protein stability of the latter, thereby enhancing glycolysis in HCC cells to accelerate their malignant progressions. These findings provide a theoretical basis for identifying the therapeutic targets for HCC.

## Conclusions

This study revealed that the expression of DNAAF5 was increased in hepatocellular carcinoma and negatively correlated with the prognosis of patients. It underlying mechanism showed that DNAAF5 directly binds PFKL and recruits the deubiquitinated protein (USP39) to improve the stability of the PFKL protein, thus enhancing abnormal glycolysis in HCC cells. Overall, we have elucidated the significance of DNAAF5 in HCC and provided a theoretical basis for subsequent studies and clinical transformation.

## Data availability statement

The original contributions presented in the study are included in the article/supplementary material. Further inquiries can be directed to the corresponding authors.

## Ethics statement

Approval of the research protocol by an Institutional Reviewer Board: Institutional Research Ethics Committee of Taizhou People’s Hospital. All animal experiments were approved by the Institutional Animal Care and Use Committee of Nantong University.

## Author contributions

HH and JH conceived the design of the study, and YL performed the most experiments. GH drafted the manuscript, and QW and TS contributed to the analysis of the data and Interpretation of section results. All of the authors have read and approved the final manuscript.

## Funding

This project was supported by Scientific research start-up fund of Taizhou People’s Hospital (QDJJ202106), Six Peak Talent Projects in Jiangsu Province (WSW-264), Taizhou “311 Project” (RCPY201813) and Taizhou Development Project (TS202001).

## Conflict of interest

The authors declare that the research was conducted in the absence of any commercial or financial relationships that could be construed as a potential conflict of interest.

## Publisher’s note

All claims expressed in this article are solely those of the authors and do not necessarily represent those of their affiliated organizations, or those of the publisher, the editors and the reviewers. Any product that may be evaluated in this article, or claim that may be made by its manufacturer, is not guaranteed or endorsed by the publisher.
