# Supplementary material for: DNAAF5 promotes hepatocellular carcinoma malignant progression by recruiting USP39 to improve PFKL protein stability
Source: Front Oncol. 2022 Oct 6;12:1032579. doi: 10.3389/fonc.2022.1032579 (PMC9582515; doi:10.3389/fonc.2022.1032579)
Supplement: Supplementary file 1 [file DataSheet_1.docx]

**DNAAF5 promotes hepatocellular carcinoma malignant progression by recruiting USP39 to improve PFKL protein stability**

Yaping Liu^1^, Qiong Wu^2^, Tiantian Sun^3^, Gaohua Han^1,*^, Hexu Han^4,*^

1. Department of Oncology, The Affiliated Taizhou People's Hospital of Nanjing Medical University, Taizhou, Jiangsu 225300, People's Republic of China
2. Department of Geriatrics, The Affiliated Taizhou People's Hospital of Nanjing Medical University, Taizhou, Jiangsu 225300, People's Republic of China
3. Medical department, The Affiliated Taizhou People's Hospital of Nanjing Medical University, Taizhou, Jiangsu 225300, People's Republic of China
4. Department of Gastroenterology, The Affiliated Taizhou People's Hospital of Nanjing Medical University, Taizhou, Jiangsu 225300, People's Republic of China

*Correspondence should be addressed to Hexu Han, Department of Gastroenterology, The Affiliated Taizhou People's Hospital of Nanjing Medical University, Taizhou, Jiangsu 225300, People's Republic of China. E-mail: [1920716577@qq.com](mailto:1920716577@qq.com) Tel.: +86 0523 89890389

**
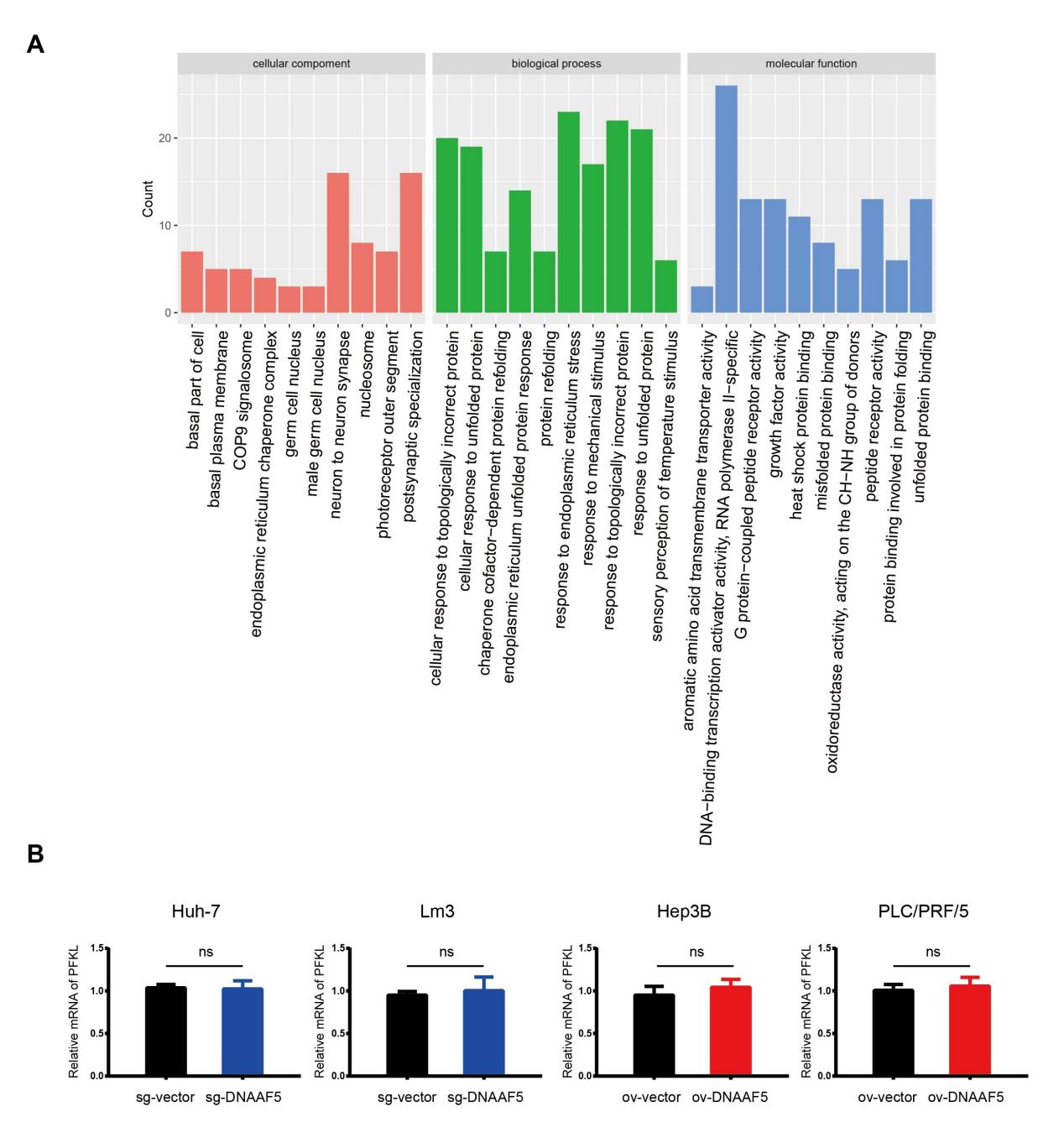
**

**Supplementary Figure 1**. DNAAF5 promotes hepatocellular carcinoma malignant progression by recruiting USP39 to improve PFKL protein stability.

1. Transcriptome sequencing results showing that DNAAF5 might exert multiple biological effects in HCC cell lines.
2. qPCR assays showed that DNAAF5 did not affect the mRNA content of PFKL in HCC cells.
